# Habenula as a Neural Substrate for Aggressive Behavior

**DOI:** 10.3389/fpsyt.2022.817302

**Published:** 2022-02-17

**Authors:** Flavia Venetucci Gouveia, George M. Ibrahim

**Affiliations:** ^1^Neuroscience and Mental Health, Hospital for Sick Children Research Institute, Toronto, ON, Canada; ^2^Division of Neurosurgery, The Hospital for Sick Children, Toronto, ON, Canada; ^3^Division of Neurosurgery, Department of Surgery, University of Toronto, Toronto, ON, Canada; ^4^Institute of Biomedical Engineering, University of Toronto, Toronto, ON, Canada; ^5^Institute of Medical Science, University of Toronto, Toronto, ON, Canada

**Keywords:** habenula, aggressive behavior (AB), neuropsychiatric symptoms, preclinical studies, review

## Abstract

Over the past decades, an ever growing body of literature has explored the anatomy, connections, and functions of the habenula (Hb). It has been postulated that the Hb plays a central role in the control of the monoaminergic system, thus influencing a wide range of behavioral responses, and participating in the pathophysiology of a number of psychiatric disorders and neuropsychiatric symptoms, such as aggressive behaviors. Aggressive behaviors are frequently accompanied by restlessness and agitation, and are commonly observed in patients with psychiatric disorders, intellectual disabilities, and neurodegenerative diseases of aging. Recently, the Hb has been explored as a new target for neuromodulation therapies, such as deep brain stimulation, with promising results. Here we review the anatomical organization of the habenula and discuss several distinct mechanisms by which the Hb is involved in the modulation of aggressive behaviors, and propose new investigations for the development of novel treatments targeting the habenula to reduce aggressive behaviors.

## Introduction

The habenula (Hb) is an epithalamic structure that presents rich connections with several cortical and subcortical structures, including the limbic system, and areas responsible for the production and regulation of monoamines (i.e., raphe nuclei for serotonin, ventral tegmental area and substantia nigra for dopamine, and locus coeruleus for noradrenaline) (1–5). These connections place the Hb in a central position for the regulation of motivated behaviors, and thus has been implicated in the pathophysiology of several disorders, such as autism spectrum disorder (ASD) (6), depression (7, 8), bipolar disorder (8–10), and schizophrenia (10, 11), as well as neuropsychiatric symptoms, such as aggressive behaviors (12, 13).

Aggressive behaviors can be verbal and physical insults directed toward oneself (i.e., self-injury behavior), others, or objects (14), and are highly correlated with restlessness and excessive motor agitation (15, 16). Several distinct classifications of human aggressive behavior have been proposed, with the classification in proactive (also known as premeditated aggression) or reactive (also known as impulsive aggression) being widely accepted (17–21). While proactive aggression is believed to involve planned behaviors to achieve a specific goal, reactive aggression is unrelated to a specific goal, being mainly associated with frustration, provocation or stress. Another important difference between these two types of aggressive behavior, is the association with high levels of autonomic arousal and impulsivity in subjects presenting with reactive aggression, that is absence in the proactive aggression (17–21). Aggressive behaviors, mainly reactive aggression, are frequent among patients with psychiatric conditions, especially in those suffering from intermittent explosive disorder, borderline/antisocial personality disorders, patients with neurodevelopmental conditions, such as ASD (17, 22), and those with neurodegenerative diseases of aging (e.g., Alzheimer's disease) (23–25).

The neurocircuitry underlying aggressive behaviors include prefrontal cortical regions and areas of the mesolimbic system, especially the hypothalamus, amygdala and periaqueductal gray matter (14–17, 26). It is believed that decreased serotonergic transmission in the prefrontal cortex reduces the top-down inhibitory control over the limbic system, resulting in motor activation and hormonal production, preparing the organism for a fight-or-flight situation (17, 21, 26). However, simplistic this mechanism might seem, there are several distinct neural-pathways involved in the association of external and internal stimuli that will result in the expression of an appropriate or inappropriate aggressive behavioral response. As such, the mechanisms by which the Hb is involved in the modulation of aggressive behaviors are numerous and still not fully understood.

In this review, we explore the anatomical organization of the habenula, describe the relevant literature on the involvement of the Hb in the modulation of aggressive behaviors and discuss future perspectives and novel therapies.

### Anatomical Organization of the Habenula

**Figure 1:**
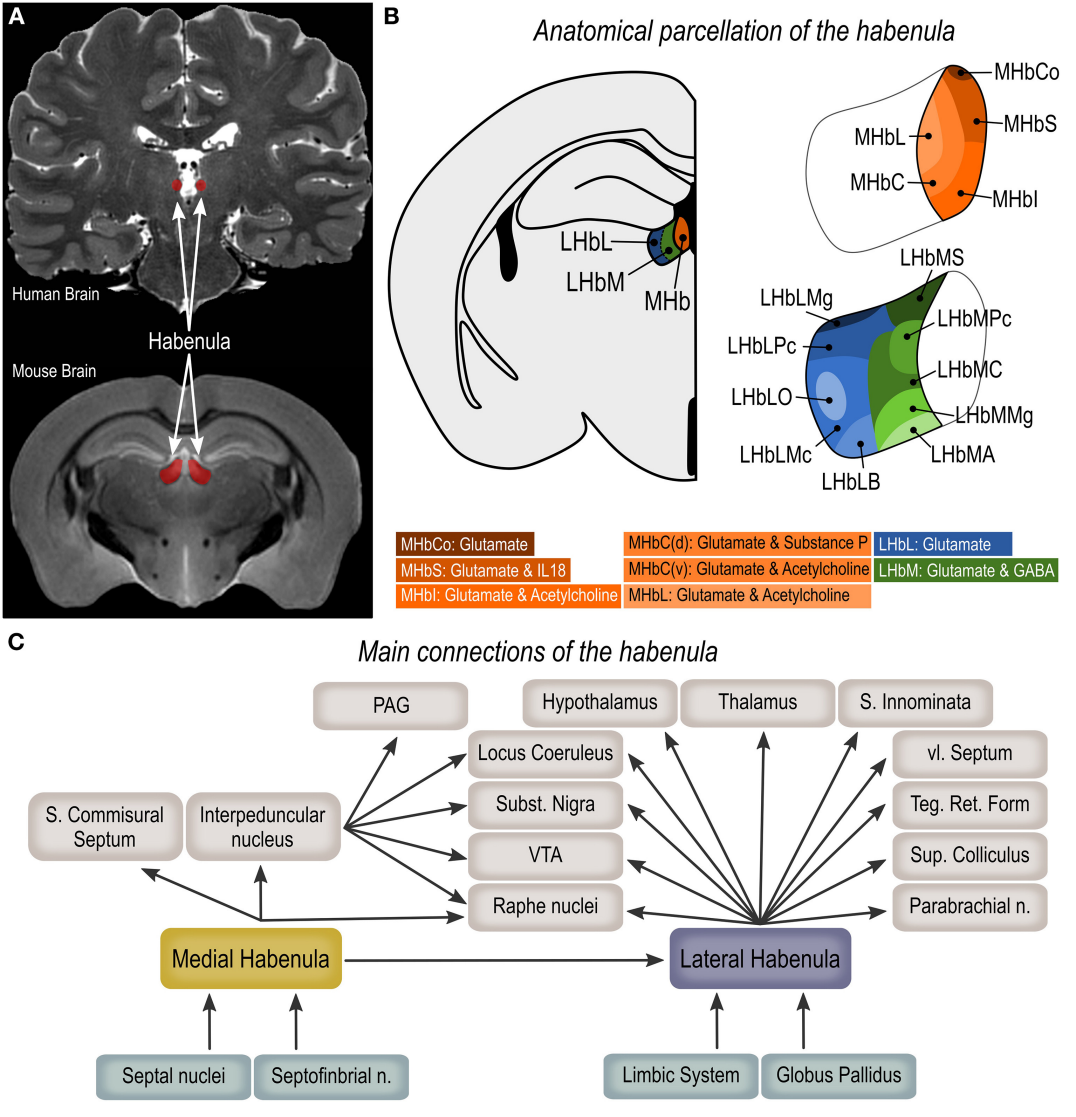
Anatomical organization of the habenula. **(A)** Magnetic resonance imaging (coronal plane) showing the human and mouse habenula (https://openneuro.org/datasets/ds002179/versions/1.1.0). **(B)** Anatomical parcellation of the medial (MHb) and lateral habenula (LHb). **(C)** Main habenula connections. MHbS, medial habenula superior part; MHbI, medial habenula inferior part; MHbC, medial habenula central part; MHbL, medial habenula lateral part; MHbCo, medial habenula commissural part; LHbMA, lateral habenula medial part anterior subregion; LHbMS, lateral habenula medial part superior subregion; LHbMPc, lateral habenula medial part parvocellular subregion; LHbMC, lateral habenula medial part central subregion; LHbMMg, lateral habenula medial part marginal subregion; LHbLPc, lateral habenula lateral part parvocellular subregion; LHbLMc, lateral habenula lateral part magnocellular subregion; LHbLO, lateral habenula lateral part oval subregion; LHbLB, lateral habenula lateral part basal subregion; LHbLMg, lateral habenula lateral part marginal subregion; PAG, periaqueductal gray matter; VTA, ventral tegmental area.

The Hb is a bilateral, phylogenetically old, epithalamic structure surrounded by the third ventricle and the thalamus (lateral and dorsal borders), the posterior commissure (ventral and posterior borders), and the stria medullaris of the thalamus (anterior limit, Figure 1A) (1, 4, 9). In mammals, the Hb is divided into two sub-regions—the medial habenula (MHb) and the lateral habenula (LHb)—based on their cellular and genetic profiles, neuroanatomical connectivity, and associated functions (Figure 1B) (1, 4, 5). Through the fasciculus retroflexus, both MHb and LHb project to distinct brain areas. MHb efferents form the core of the fiber bundle that reaches the interpeduncular nucleus (IP) in a 90° rotation pattern, with dorsal projections reaching the lateral aspect of the IP, medial projections to the ventral aspect of IP and the lateral projections ending on the dorsal aspect of IP (2). Projections from the IP then reaches the periaqueductal gray matter (27), an area critically involved in the neural network of aggressive behavior (17, 21, 26). Discrete projections from the MHb can also be found in the LHb, supra-commissural septum and median raphe nucleus (2, 28). Inputs form the medial, lateral and triangular septal nuclei and septofimbrial nucleus, *via* the medial stria medullaris comprise the main afferent projections to the MHb (2, 5, 29). LHb efferents forms the mantle portion of the fasciculus retroflexus, that reaches the ventral tegmental area, hypothalamus (i.e., lateral, posterior and dorsomedial hypothalamic nuclei, lateral preoptic area), ventromedial thalamic nucleus, substantia innominata, ventrolateral septum, substantia nigra pars compacta, medial and dorsal raphe nuclei, and tegmental reticular formation (2). Discrete additional projections can be found in the pretectal area, superior colliculus, nucleus reticularis tegmenti pontis, parabrachial nuclei, and locus coeruleus (2). Afferent projections from limbic regions are mainly found in the more medial aspect of the LHb while projections from the globus pallidus reach the lateral aspect of the LHb (Figure 1C) (2).

#### Medial Habenula

The MHb can be further divided into five subregions, namely superior (MHbS), inferior (MHbI), central (MHbC), lateral (MHbL), and commissural (MHbCo) parts (Figure 1B). The MHbS consists exclusively of densely packed glutamatergic neurons that strongly express interleukin-18, with nuclei on a typical triangular appearance, thin dendrites and tightly packed synaptic vesicles in axon terminals (3, 30). The cell characteristics of neurons in the MHbI are similar to the one in the MHbS, however the nuclei are typically round and the proximal dendrites are thicker. Also, these neurons are not exclusively glutamatergic as they co-transmits acetylcholine from the axonal terminals (3, 30). Likewise, the MHbL is also composed of cholinergic and glutamatergic neurons, however these are smaller, with oval nuclei and nuclear membrane surrounded by nucleoli or chromatin plaques (3, 30). The MHbC can be viewed as a transition area, as it is composed of a combination of cell clusters composed of the diverse cell types observed in the adjacent regions, that are separated from each-other by the terminal fiber bundles of the stria medullaris. While the dorsal region of the MHbC is composed of neurons that co-express substance-P and glutamate, the ventral region is both cholinergic and glutamatergic (3, 30). Finally, the MHbCo displays the largest glutamatergic neurons in the MHb area, with unusual nuclei in a semilunar shape (3, 30).

#### Lateral Habenula

The LHb is significantly larger than the MHb and is subdivided into medial (LHbM) and lateral (LHbL) parts (Figure 1B). Each of these aspects are further subdivided resulting in a total of ten LHb subregions (3). Irrespective of cell morphology and location, neurons in the LHb are predominantly glutamatergic (30), however it has recently been shown that a very discrete population of GABAergic neurons are present in the medial part of the LHb (31, 32). The LHbM is divided in anterior (LHbMA), superior (LHbMS), parvocellular (LHbMPc), central (LHbMC), and marginal (LHbMMg) subregions. Small neurons are found in the LHbMA, LHbMMg and LHbMPc parts, with LHbMA showing round multipolar cells with heavily folded nuclei, LHbMMg having an elongated version of the LHbMA cells, and LHbMPc presenting a spindle-shaped cell with deeply invaginated nucleus. Greatly larger neurons are found in the LHbMS and LHbMC subregions, however LHbMS neurons are characterized by an oval perikarya, and LHbMC cell nuclei are often invaginated and display a brighter and finer karyoplasm (3). The LHbL is divided into parvocellular (LHbLPc), magnocellular (LHbLMc), oval (LHbLO), basal (LHbLB), and marginal (LHbLMg) subregions (3). Neurons in the LHbLPc and LHbLMg parts are predominantly small to medium-sized. Cells in the LHbLMc, and LHbLO are large, and while neurons in the LHbLMc and LHbLB parts present thick and long dendrites, neurons in the LHbLO extend thin ones (3).

### Habenula as a Key Relay for Aggressive Behavior

Several clinical and preclinical studies have investigated the involvement of the Hb in the modulation of aggressive behaviors. Studies using transgenic models have provided further evidence of the involvement of the Hb in the regulation of aggressive behaviors. Using double and triple transgenic zebrafish, Chou et al. (33) demonstrated that the dorsal habenula–interpeduncular nucleus pathway [homologous to the mammalian MHb-IP pathway (34)], is key for the modulation of aggressive behaviors, with connectivity between the medial subregion of dHb and IP being associated with increase aggression (33). GPR3 is an G-protein-coupled-receptor broadly expressed in the central nervous system, with maximal expression in the Hb, that has been implicated in the regulation of cAMP signaling and, consequently, modulation of emotional behavior. Knockout Gpr3^−/−^ mice present null expression of GPR3 in the Hb, high levels of aggressive behavior and accentuated reduction of serotonin, noradrenaline and its metabolites in the hypothalamus and frontal cortex (35). The Disrupted-In-Schizophrenia-1 (DISC1-Q31L) mouse model of depression, bipolar disorder and schizophrenia, presents heightened inter-male aggressive behavior along with increased neuronal density in both the LHb and MHb (36). On the other hand, male Mecp2^−^ mice (i.e., knockout of the X-linked methyl-CpG-binding protein 2, gene associated to Rett syndrome) present absence of aggressive behavior and accentuated reduction in oxytocinergic innervation in the lateral habenula (37). It is important to highlight, however, that transgenic animals may also present with additional brain alterations in function and connection and, thus, the altered behavior observed in these studies may be the result of the sum of all these changes and not rely solely on alterations observed in the habenula.

In lactating females, aggressive behaviors toward an intruder are mediated by the medial prefrontal cortex-LHb-dorsal raphe nucleus pathway, as demonstrated by increased co-labeling of c-Fos- and Fluorogold-positive neurons in the mPFC and LHb following aggressive encounters (38). Pharmacological manipulation of NMDA and AMPA/kainate receptors *via* microinjection of receptor antagonists in the dorsal raphe nucleus of lactating females is capable of inhibiting this behavior (38). Interestingly, injection of arginine-vasopressin V1a receptor antagonists in the LHb or dorsal raphe nucleus, of both male and female mice, is not sufficient to alter aggressive behaviors, suggesting that the arginine-vasopressin system does not play a crucial role in this neurocircuitry (39). Tear fluid is rich in pheromones capable of eliciting several context-specific behavioral responses in both males and females rodents (40). It has been shown that female mouse tears suppress aggressive behaviors in males and induce a great increase in c-Fos immunoreactivity in the medial aspect of the LHb (41).

A study investigating habenula resting-state functional connectivity in highly reactive aggressive men showed an association between high levels of trait aggression to lower global efficiency of the left habenula and atypical habenula-prefrontal connectivity (42). In a recent work from our group, we showed that the Hb—along with the dorsal raphe nuclei, substantia nigra, ventral tegmental area, and locus coeruleus—is part of the functional connectivity map associated with symptom alleviation in a patient treated with deep brain stimulation of the posterior hypothalamus for reduction of severe and treatment refractory aggressive behavior (43).

#### Reward Value of Aggressive Behavior

Another line of evidence is based on the well-known involvement of the LHb in reward-related behaviors. The suppression of midbrain dopaminergic neurons, *via* GABAergic indirect connections, is thought to be the main mechanism by which the LHb drives reinforcement learning (1). The LHb is active in response to the negative value of a stimulus, unexpected reward omissions, and cues associated with these stimuli (44, 45), such as in situations of drug withdrawal (46–48). Moreover, the functional integrity of the LHb is necessary to integrate proactive and retroactive information to guide behavioral flexibility when the reward contingencies change (49). Golden et al. (50) investigated the involvement of basal forebrain projections to the LHb in the modulation of aggression reward. Using a conditioned place preference (CPP) or place aversion (CPA) paradigm, the authors have shown that aggressive mice presented CPP for the chamber where aggressive encounters occurred, while non-aggressive mice developed a CPA to the same chamber. Using optogenetic techniques to stimulate or inhibit the basal forebrain projections to the LHb, it was found that the stimulation of these terminals promotes CPP and reduces LHb firing, and the inhibition results in CPA and increases LHb firing (50). These results are in line with previous findings indicating that decreased LHb activity is associated with rewarding components of behavior. Flanigan et al. (32) showed that optogenetic stimulation of orexin terminals located in GABAergic neurons within the LHb promotes inter-male aggressive behavior and CPP for aggression-paired contexts.

The reward value of aggressive behaviors is also mediated *via* the LHb-ventral tegmental area (VTA)-nucleus accumbens (nAcc) network that results in increased dopamine release in the nAcc when animals are expecting a conditioned aggressive encounter (51). Moreover, antagonism of both dopamine receptor types 1 and 2 in the nAcc have been described as reducing the rewarding value of aggressive behaviors (52–54). In line with these findings, two case reports (55, 56) and one case series (57) reporting on patients treated with deep brain stimulation of the nAcc for severe refractory aggressive behaviors, have shown long lasting positive results (58).

#### The Circadian Cycle and Aggressive Behavior

There is a strong body of evidence on the role of the Hb in regulating the circadian cycle, thus influencing internal physiology, brain activity patterns and day-night behavioral rhythm (59–62). Anatomically, the Hb shares the epithalamus with the pineal gland, a brain structure responsible for the production of the hormone melatonin, that serves among others, as a major regulator of the sleep-wake cycle (5, 63). Similar to the pineal gland, the Hb expresses mRNA for arylalkylamine-N-acetyltransferase, the enzyme responsible for melatonin synthesis, thus being implicated as a supplementary location for melatonin biosynthesis (63). Dysregulations of the circadian cycle are known to negatively influence cognition, emotions and behavior, and is associated with worsening of symptoms in several neuropsychiatric disorders (64, 65). Poor sleep routines (i.e., short sleep duration, inadequate sleep quality) in children and adolescents is associated with increased irritability, conduct problems, anxiety, and hyperactivity (66–68), and in adults, is associated with increased hostility, anger, aggression and suicidal ideation (49–52). However, it is important to highlight that the Hb circadian clock is independent of the suprachiasmatic nucleus (60), and although disruption of the Hb circadian clock does not disrupt the sleep cycle, it is capable of altering the subjects response to stressors, suggesting that a sleep-independent effect on aggression may exist (69).

Among the various neurotransmitters, neuropeptides, and neurohormones involved in the modulation of the circadian cycle, dysfunctions in serotonin transmission are thought to be central in the association between poor sleep and aggressiveness. *Via* GABAergic interneurons, the LHb modulates the activity of serotonergic neurons in the dorsal raphe nucleus (70, 71), that have an increased activity during wakefulness, along with brain-wide increase in serotonin levels (72, 73). The prolonged exposure to high serotonin levels caused by reduced sleep time causes gradual desensitization of serotonin receptors (74), thus contributing to a reduced serotonergic effective transmission in the prefrontal cortex and consequently reduction in the top-down inhibitory control of emotions described above.

## Discussion

Excessive aggressive behaviors are highly prevalent, particularly among patients with psychiatric disorders and presents a major obstacle for patient care, increasing institutionalization rates and reducing patients' quality of life (15, 17). The clinical and preclinical studies described here show evidence of the involvement of the habenula in the neuro-circuitry of aggressive behavior, and suggest that the modulation of neurons in this area could result in symptom alleviation in patients presenting psychiatric disorders associated with aggressive behavior. Furthermore, typical and atypical antipsychotics and antidepressants are commonly used for the treatment of aggressive behaviors *via* antagonizing dopaminergic receptors or selectively inhibiting serotonin reuptake enzymes (17). As described above, the Hb is highly connected with areas responsible for the production and regulation of monoamines (e.g., dopamine and serotonin) and thus, involved in the response to standard treatments (1–5). However, the chronic systemic exposure to these compounds is associated with refractoriness to treatment, and may produce severe side effects that can escalate to the point of being impeditive of treatment (14, 16, 75, 76). Thus, further studies are necessary to better understand the brain mechanisms associated with aggressive behaviors and develop novel treatments that are tailored to safely and effectively improve patient outcomes.

Focused ultrasound is being intensively investigated as a novel non-invasive tool for neuromodulation that could be used to deliver pharmacological agents to localized brain areas, without the need of a systemic distribution (77). By repurposing a commercially available ultrasound contrast, Lea-Banks and colleagues fabricated an ultrasound-sensitive nanodroplet loaded with or without anesthetic drug, that was injected intravenously and then vaporized in a discrete brain target using focused ultrasound (78, 79). The authors showed that the use of the unloaded nanodroplets increased local neuronal activity, while drug-loaded nanodroplets suppressed (78, 79). Considering that the Hb is involved in the regulation of monoamines, such as those modulated by drug therapy, and the current evidence on the possibility of reducing the reward value of aggressive behavior by increasing neuronal firing in the LHb, one could envision further exploring this innovative technique to selectively deliver nanodroplets to the LHb and modulate its activity, to reduce aggressive behavior in patients with psychiatric disorders that do not present adequate response to conventional therapy.

Deep brain stimulation is a neuromodulation therapy that involves the precise placement of electrodes into deep brain structures to modulate neuronal activity *via* the application of an electrical current that can be precisely titrated (80). Although only a few case studies on Hb deep brain stimulation have been published, they report beneficial outcomes in patients suffering from schizophrenia (81), depression (82, 83), obsessive-compulsive disorder (84), and bipolar disorder (85). New clinical trials are currently being performed, demonstrating a growing interest to target this region for the treatment of psychiatric disorders [for a review on deep brain stimulation of the habenula see (86)]. Considering the strong evidence on the involvement of the Hb in the pathophysiology of aggressive behaviors and the possibility of safely targeting this area with deep brain stimulation, it would be interesting to investigate Hb deep brain stimulation in the context of aggressive behaviors.

In this article we provide a detailed review of the anatomical organization of the Hb, by describing cell characteristics and connections of the lateral and medial aspects of the Hb and its subdivisions. We discussed several distinct mechanisms by which the Hb modulates aggressive behavior, detailing studies investigating transgenic models, neuronal modulation and neuroimaging, and the literature about the involvement of the Hb in reward-related behaviors and regulation of circadian cycle. We concluded this review discussing how innovative neuromodulatory techniques could be investigated in the context of Hb and aggressive behaviors to improve patient outcome.

## Author Contributions

Both authors listed have made a substantial, direct, and intellectual contribution to the work and approved it for publication.

## Conflict of Interest

The authors declare that the research was conducted in the absence of any commercial or financial relationships that could be construed as a potential conflict of interest.

## Publisher's Note

All claims expressed in this article are solely those of the authors and do not necessarily represent those of their affiliated organizations, or those of the publisher, the editors and the reviewers. Any product that may be evaluated in this article, or claim that may be made by its manufacturer, is not guaranteed or endorsed by the publisher.
